# Epithelial ovarian cancer stem-like cells expressing α-gal epitopes increase the immunogenicity of tumor associated antigens

**DOI:** 10.1186/s12885-015-1973-7

**Published:** 2015-12-16

**Authors:** Xiaofen Yao, Zhangli Dong, Qiuwan Zhang, Qian Wang, Dongmei Lai

**Affiliations:** The Center of Research Laboratory, and Department of Gynecology, The International Peace Maternity and Child Health Hospital, School of Medicine, Shanghai Jiaotong University, Shanghai, China

**Keywords:** Ovarian cancer, α-gal epitopes, Immunotherapy, Ovarian cancer stem-like cells, Protein c-erbB-2

## Abstract

**Background:**

As ovarian cancer stem cells (CSCs) are responsible for tumor initiation, invasion, metastasis, and chemo-resistance, new stratagems that selectively target ovarian CSCs are critically significant. Our previous work have demonstrated that ovarian cancer spheroid cells are tumorigenic and chemo-resistant, and have the properties of ovarian CSCs. Herein, we hypothesized that expressing α-gal epitopes on ovarian spheroid cells may help eliminate CSCs and improve the outcome of therapeutic intervention for ovarian cancer patients.

**Methods:**

Lentivirus-mediated transfer of a pig α(1,3)galactosyltransferase [α1,3GT] enzyme gene into human ovarian cell line SKOV3 cells formed α-gal epitope-expressing cells (SKOV3-gal cells), and then these cells were maintained in a serum-free culture system to form SKOV3-gal spheroid cells. Efficacy of this cell vaccine was demonstrated in α1,3GT knockout mice (α1,3GT KO mice).

**Results:**

The antibody titers to α-gal epitopes measured by ELISA were significantly increased in α1,3GT KO mice after immunization with SKOV3-gal spheroid cells. Furthermore, compared with the non-immunized KO mice, the SKOV3 tumors grafted under renal capsules of KO mice immunized with SKOV3-gal spheroid cells grew slower and began to shrink on day 12. Western blot analysis also showed that immunized KO mice can produce effective antibody against certain tumor associated antigens (TAAs) derived from both SKOV3 cells and SKOV3 spheroid cells. The TAAs were further investigated by mass spectrometry and RNA interference (RNAi) technology. The results suggested that antibodies responding to protein c-erbB-2 may be raised in the sera of the mice after immunization with SKOV3-gal spheroid cells. Ultimately, vaccination with SKOV3-gal spheroid cells induced more CD3 + CD4 + T cells in the spleen of immunized mice than non-immunized KO mice.

**Conclusions:**

The results suggest that vaccination using ovarian cancer stem-like cells engineered to express α-gal epitopes may be a novel strategy for treatment of ovarian cancer.

**Electronic supplementary material:**

The online version of this article (doi:10.1186/s12885-015-1973-7) contains supplementary material, which is available to authorized users.

## Background

Epithelial ovarian cancer (EOC) is the most lethal gynecological malignancy. Most ovarian cancer patients are treated with cytoreductive surgery followed by combination chemotherapy. Although the majority of patients respond to first-line treatment, most will relapse and overall survival rates for patients with advanced EOC remain disappointing [1–3]. As such, it remains critical to develop an effective therapy to reduce the relapse and achieve long-term remissions.

In recent years, the study of cancer stem cells (CSCs) has resulted in an improved understanding of carcinogenesis. CSCs are proposed to represent the fundamental driving force of tumor development, initiation, invasion, and metastasis, as well as recurrence [4]. Conventional anti-cancer treatments (e.g. chemotherapy and radiation) can often transiently shrink tumors, but these therapies fail to target and kill CSCs, leading to relapse, and ultimately death [5]. As such, it is necessary to develop an effective stratagem targeting CSCs. Evidence to date has demonstrated that ovarian CSCs are tumorigenic and chemo-resistant. Elimination of ovarian CSCs, therefore, might help improve the sensitivity of chemotherapy drugs and reduce the tumor relapse [6, 7].

Considering that the immune system plays a pivotal role in tumorigenesis, immunotherapy has been established as an important component of many cancer treatment regimens, which could destroy cancer cells both *in situ* and within the metastatic compartment [8–10]. In addition, immunotherapy using antibodies (Abs) targeting tumor-specific antigens expressed on CSCs can selectively kill CSCs, while sparing the normal counterpart [11]. Furthermore, tumor vaccines have also showed promising preliminary data in targeting CSCs. The prerequisite for the induction of an effective antitumor immune response by tumor vaccine is the effective uptake of this vaccine by professional antigen-presenting cells (APC). It was reported that the addition of α-gal epitope to MUC1^+^ pancreatic carcinoma whole-cell vaccine could enhance presentation to APC and induce immune responses against not only differentiated cancer cells but also CSCs [12].

The α-gal epitope is a glycoconjugate present on cell membranes of non-primate mammals, prosimians and New World monkeys, but not in humans. However, the corresponding human anti-Gal antibody was found to be present in high titer in the serum of every normal individual studied [13] and is continuously produced as an immunological response to antigenic stimulation by bacteria of the normal flora [14]. It is reported that α-gal epitope specific IgG, IgM, IgD, and IgA titers remained unvaried over longer time periods in healthy subjects [15]. Tumor cells engineered to express α-gal epitopes were able to bind anti-Gal and to be destroyed by this antibody in an experimental animal model [16].

Consistent with other studies [6, 7], our previous work demonstrated that ovarian epithelial cancer cells cultured in serum-free medium could form spheroid cells, which are cancer stem-like cells that have the characterization of CSCs and can be distinguished from differentiated ovarian cancer cells [17–19]. Herein, we hypothesized that biosynthesis of α-gal epitopes to ovarian cancer spheroid cells could effectively induce Abs production against ovarian cancer stem-like cells. Using α1,3GT knockout mice, we further investigated the immune response induced by vaccines expressing α-gal epitopes against both differentiated ovarian cancer cells and cancer stem-like cells.

## Methods

### Cell culture

All cell lines were obtained from Shanghai Cell Bank of Chinese Academy of Sciences (Shanghai, China). 293 T cells (Immortalized human embryonic kidney cells) were cultured in Dulbecco’s modified Eagle’s medium (DMEM; Gibco, Grand Island, NY) supplemented with 10 % fetal bovine serum (FBS) in a humidified incubator with 5 CO_2_ and 95 % air at 37 °C. Human ovarian cancer cell line SKOV3 cells were maintained in McCoy’s 5A medium (Sigma-Aldrich, Oakville, ON, Canada) supplemented with 10 % FBS. Then the SKOV3 cells were dissociated by 0.02 % trypsin-EDTA and maintained under stem cell conditions as described before [17–19]. In this condition, cancer cells grow as non-adherent spheroid cells. Culture media were changed every 2 days by centrifuging at 800 rpm for 5 min to remove the dead cell debris. Regular culture plates were coated with poly (2-hydroxyethyl methacrylate) (Sigma) before spheroid cell culturing [17–19]. 293 T cells were used for recombinant lentivirus transfection, amplification, and titration.

### Construction of recombinant lentivirus vector expressing pig α1,3GT gene

Primers for amplification of α1,3GT coding sequence (α1,3GT CDS) were previously reported by Yu et al. [20]. Compared with the plasmid profile of pCDH-CMV-MCS-EF1-copGFP (System Biosciences, Mountain View, CA), the amplified sequence of α1,3GT CDS was analyzed for XbaI and BamHI restriction enzymes sites, and confirmed with NEBcutter V2.0. XbaI and BamHI restriction sites were designed into the primers of α1,3GT CDS prior to gene amplification. Both α1,3GT CDS and pCDH-CMV-MCS-EF1-copGFP was digested by XbaI and BamHI and the resulting 1.19 kb fragment of porcine α1,3GT cDNA was subcloned into pCDH-CMV-MCS-EF1-copGFP to construct pCDH-CMV-α1,3GT-EF1-copGFP. Finally the recombinant plasmid was used to produce lentivirus-based vector with three plasmid packaging systems including another two plasmids - pCMV dR8.91 and VSVG [21]. All three plasmids were co-transfected into packaging 293 T cells using the calcium phosphate method [21]. High titers of recombinant lentivirus were amplified, purified, and stored. Eventually SKOV3 cells were transfected with lentivirus vector at multiplicity of infection (MOI) of 40, which formed SKOV3-gal cells expressing α-gal epitopes.

### Real-time quantitative PCR

After total RNA was isolated from the samples, five hundred nanograms of total RNA from each sample were reverse transcribed using the primescript RT reagent kit (Takara Bio Inc., Shiga, Japan). Real-time PCR was performed on cDNA using SYBR Premix Ex Taq (Takara) on the Mastercycler® ep realplex (Eppendorf, Hamburg, Germany). All reactions were carried out in triplicate using a 25-μl volume. In brief, PCR amplification was carried out using an initial denaturation at 95 °C for 5 min, followed by 40 cycles for 30 s at 95 °C, 30 s at 60 °C, and 30 s at 72 °C. A sample lacking template DNA was used as a negative control. The primers for the genes are provided in Additional file 1: Table S1.

### Proliferation and drug resistance assessment

SKOV3 cells and SKOV3-gal cells were seeded at a density of 3 × 10^3^ cells per well in 96-well plates. The MTT assay was employed to count the viable number of cells at 1, 2, 3, 4, 5, and 6 days after seeding cells. A growth curve was plotted for each cell line depicting the viable number of cells versus the duration post infection.

To assess the effect of α-gal epitopes on drug resistance, SKOV3 cells and SKOV3-gal cells were plated in culture medium containing cisplatin (Sigma-Aldrich, 20 μmol) for 24 h and 48 h. Cultures were set up in triplicate. The proliferation conditions were determined by MTT assay with an OD reading at 490 nm. Each experiment was repeated three times under the same experimental conditions.

### Complement-mediated cell death

Both SKOV3 cells and SKOV3-gal cells were incubated in the presence of 1:3, 1:6, 1:9, and 1:18 human serum solution diluted with McCoy’s 5A medium without FBS for twenty-four hours. Human blood samples were collected from three ovarian cancer patients who were categorized as stage III, grade 2–3 serous adenocarcinoma according to the International Federation of Gynecology and Obstetrics (FIGO) classification. The present study was approved by the institutional review boards at Shanghai Jiaotong University and written informed consent was obtained from these patients. SKOV3 cells and SKOV3-gal cells without human serum and FBS were used as negative controls. McCoy’s 5A medium without FBS was used as blank control. Cultures were set up in triplicate. Proliferation was monitored by MTT assay under optical density (OD) reading at 490 nm. The percentage of survival rate was determined as follows: (OD_490(sample)_- OD_490(blank control)_)/(OD_490(negative control)_- OD_490(blank control)_) × 100 %.

### Antibody-dependent cell-mediated cytotoxicity (ADCC) assay

The ADCC assay was performed as described by Tanemura et al. [22] with minor modifications. SKOV3 cells and SKOV3-gal cells were plated in 96-well plates. Subsequently, peripheral blood mononuclear cells (PBMC) were obtained by Ficoll–Hypaque centrifugation of venous blood from ovarian patients and were added to the wells at cell ratios of 1:20 (effector cells: target cells) in the presence or absence of serum from the same patient. The plates were incubated at 37 °C for 72 h and MTT assay was employed to count the number of viable cells. The percentage of cytotoxicity rate was determined as follows: 1-(OD_490(sample)_-OD_490(PBMC)_)/OD_490(target cells)_.

### Tumor cell vaccination

Six α1,3GT knockout mice were obtained from Dr Jung-Ah, Cho (Center for animal resource development, College of Medicine, Seoul National University, Seoul, Korea). The genotypes were confirmed by polymerase chain reaction (PCR) on DNA extracted from the tail of the α1,3GT KO mice [23], with the wild-type (WT) mice counterpart (C57BL/6) as control. The mice were mated to supply enough mice for the experiment. Prior to the experimental procedure, six-week-old KO mice received 4-weekly intraperitoneal immunization of 100 mg of pig kidney membrane homogenate to induce high titers of anti-Gal Abs as described previously in humans [22, 24]. Subsequently, the KO mice were vaccinated subcutaneously three times at 1 week intervals with 1 × 10^6^ 50 Gy–irradiated SKOV3 cells, SKOV3 spheroid cells, SKOV3-gal cells, or SKOV3-gal spheroid cells. One week after vaccination, the mice were assessed for the immune response as described below. All animal procedures were approved by the Institutional Animal Care and Use Committee of Shanghai, and were performed in accordance with the National Research Council Guide for Care and Use of Laboratory.

### Enzyme-linked immunosorbent assay (ELISA)

The production of anti-Gal IgG antibody was measured by ELISA. Galalph1-3Gal beta1-4GlcNAc-BSA (Dextra Laboratories, Reading, UK) was attached to microtiter plates at a concentration of 10 μg/ml in carbonate buffer, pH 9.5. The plates were coated at 4 °C overnight. After washing five times, 1 % bovine serum albumin in carbonate buffer was added to the wells to prevent nonspecific binding. The serum samples obtained from vaccinated-KO mice were added at various dilutions with PBS containing 0.3 % BSA. One hour later, horseradish peroxidase (HRP)-linked goat anti-mouse IgG was added. A color reaction was obtained with peroxidase reagent 3.30, 5.50-tetramethylbenzidine (TMB, Sigma-Aldrich), and stopped by the addition of 2 M H_2_SO_4_. The optical density was read at 450 nm using a Bio-Rad ELISA reader.

### In vivo studies of tumor cell vaccine in an experimental mouse model

5 × 10^6^ SKOV3 cells were injected subcutaneously into 6 to 8 week-old female (*n* = 8) athymic nude mice to form SKOV3 tumors. After injection, the nude mice were kept in a specific pathogen-free environment and checked for tumor development every two days for 1 month. Simultaneously, the α1,3GT KO mice were pre-immunized with pig kidney fragments and then vaccinated with 50 Gy–irradiated SKOV3 cells, SKOV3 spheroid cells, SKOV3-gal cells, or SKOV3-gal spheroid cells as described above. One week after the last immunization, the tumors formed in nude mice were collected and stored at 4 °C in Hanks’ balanced salt solution supplemented with antibiotics. Within 2 h, the tumor sample was cut into two parts. One part was fixed for histological analysis and the other was cut into multiple 1 mm^3^ pieces, which were meticulously implanted under the renal capsule of the left kidney (2 grafts per kidney) of the immunized α1,3GT KO mice (*n* = 15 in each group) as described previously by Lee et al. [25]. Subsequently, on days 4, 8, and 12 after grafting, five mice in each group were euthanized under anesthesia and the kidneys were removed and the grafts were measured *in situ* by using a stereoscopic microscope, fitted with an ocular micrometer, and calibrated in ocular units (OMU, 1OOMU = 1 mm) to examine tumor growth. Two perpendicular diameters were measured and the difference in mean tumor diameter was calculated [26].

### Western blotting analysis

The production of Abs toward tumor antigens on SKOV3 cells or SKOV3 spheroid cells was examined by Western blot analysis using vaccinated KO mice sera. The total amount of proteins from the cultured cells was subjected to 10 % SDS-PAGE (50 μg protein each lane) and transferred onto Hybrid-PVDF membranes (Millipore, Bedford, USA). After blocking with 10 % (w/v) non-fat dried milk in TBST (Tris-buffered saline containing Tween-20), the PVDF membranes were washed with TBST at room temperature and incubated with immunized α1,3GT KO mice sera diluted with 5 % (w/v) non-fat dried milk in TBST overnight. Following extensive washing, membranes were incubated with HRP-conjugated goat anti-mouse IgG secondary anti-body (1:5000; Santa Cruz Technology, Santa Cruz, USA). After washing, the immunoreactivity was visualized by enhanced chemiluminescence using ECL kit from Thermo Fisher Scientific.

To confirm whether c-erbB2 protein was existent in the 185 kDa band and 150 kDa band of the lysates of SKOV3 adherent and spheroid cells, Western-blot analysis was performed. Both 5 μl and 10 μl lysate were loaded and reacted with anti-ERBB2 antibody (Ascend Biotechnology, AR0071) and anti-β-actin antibody (Santa Cruz Technology).

### Flow cytometric (FCM) analysis

The expression of α-gal epitopes was evaluated on SKOV3-gal cells by flow cytometry. Approximately 1 × 10^6^ parental SKOV3 and α1,3GT transfected cells were suspended in 2 % BSA/PBS and incubated with biotinylated bandeiraea simplicifolia isolectin B4 (BS-IB4) lectins, which specifically binds to α-gal epitopes. Then cells were incubated with PE-Cy5 conjugated streptavidin.

The effect of cancer cell vaccine on the immune system of the KO mice was evaluated by analyzing the changes of CD3 + CD4+ and CD3 + CD8+ T cells in spleen. First the spleen was removed, then cut into pieces and grinded gently through a 200 mesh sterile nylon net. The cell suspension was carefully collected and layered on the Ficoll-Paque, PREMIUM (GE Healthcare Life Sciences, USA). The separated splenic mononuclear cells were incubated with fluorochrome-conjugated antibodies directed at the following CD markers: PE anti-mouse CD3, FITC anti-mouse CD4, and PE-Cy7 anti-mouse CD8 (all purchased from eBioscience, San Diego, CA). Gated CD3 positive events were analyzed for CD8+ and CD4+ T cells. Flow cytometry was performed using a FC500 flow cytometer (Beckman Coulter, Fullerton, CA) and analyzed using Beckman Coulter CXP software.

### Mass spectrometry analysis

Proteins were identified by mass spectrometry from bands digested from preparative gels run with 50 μg of protein on each lane and stained with commassie blue staining solution. Picking, destaining, digestion, extraction, and sample preparation of the bands were carried out. The lyophilised samples were diluted with 5 % aqueous acetonitrile (ACN) and 0.1 % formic acid and subsequently analyzed using a Thermo Finnigan LTQ ion trap mass spectrometer (Thermo Finnigan, Bremen, Germany) at the core facility of Shanghai Institutes for Biological Sciences, Chinese Academy of Sciences. Database searches were done using Sequest (TurboSEQUEST v. 27) search engines against the nonredundant IPI human database version 3.68.

### RNA interference

To knock down the ERBB2 gene expression, two small interference RNAs (siRNAs) targeting different sites of human ERBB2 mRNA (Genbank accession no. NM_004448.3) were designed and synthesized by GenePharma Co. (Shanghai, China), and a control siRNA that could not target any human mRNA was synthesized as a negative control (N.C. for short). Additionally, normal SKOV3 cells served as an untreated control (Untreated for short). The detailed information of the siRNA was listed in Additional file 2: Table S2.

One day prior to transfection, 1 × 10^5^ SKOV3 cells were seeded in six-well plates and incubated at 37 °C with 5 % CO2 overnight. The next day, cells were grown to about 50 % confluence and the medium was changed for 1.5 ml fresh McCoy’s 5A medium. Then the cells were transfected with 80 nM of siRNA using DharmaFECT reagent (Thermo Fisher Scientific Inc. MA. USA) according to the manufacturer’s instructions. Forty-eight hours after transfection, total RNA was collected for real-time PCR analysis and proteins were collected for western blot analysis. The primers for ERBB2 were as follows: 5’- CGCTTTTGGCACAGTCTACA-3’ (forward) and 5’- TCCCGGACATGGTCTAAGAG-3’ (reverse). GAPDH served as an internal control for normalization and the primers for PCR were as follows: 5’- ATGGGCAGCCGTTAGGAAAG-3’ (forward) and 5’- TGAAGGGGTCATTGATGGCA -3’ (reverse). PCR amplification was carried out using an initial denaturation at 95 °C for 2 min, followed by 40 cycles for 5 s at 95 °C and 34 s at 60 °C.

### Statistical analysis

Parametric comparisons of normally distributed values that satisfied the variance criteria were made by unpaired Student’s t-tests. Data that did not pass the variance test were compared with a nonparametric two-tailed Mann–Whitney rank sum test.

## Results

### Generation of stable ovarian cancer cell line SKOV3 transfectant expressing α-gal epitopes

**Fig. 1:**
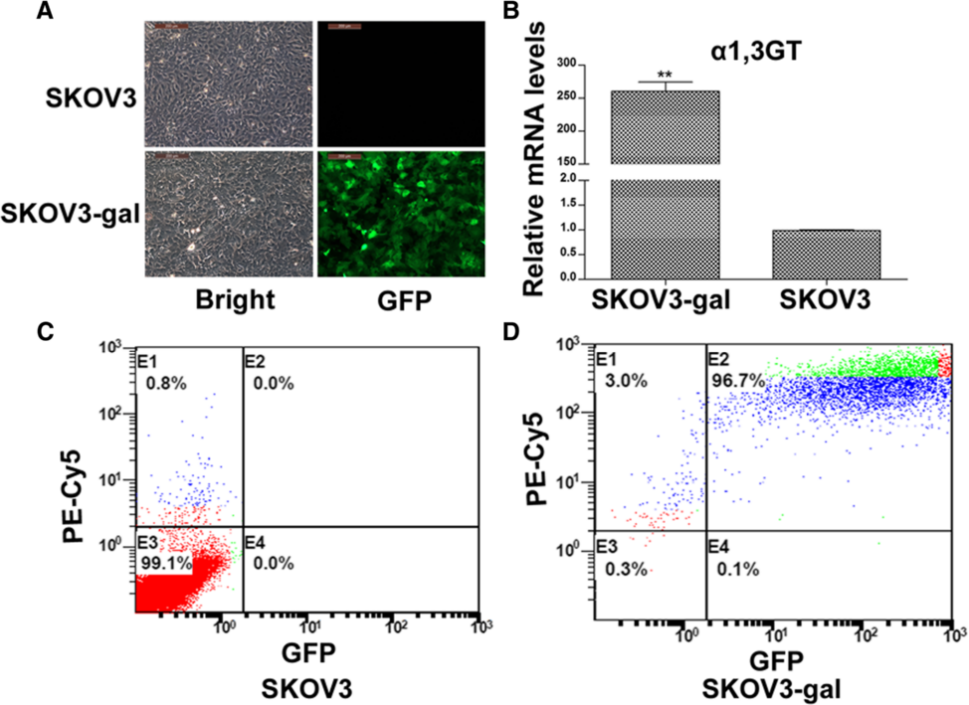
Generation of stable α-gal epitope-expressing SKOV3 cell lines. **a** Following α1,3GT expressing lentivirus infection, GFP was observed on almost all the SKOV3-gal cells under fluorescent microscopy. No GFP expression was observed on the parental SKOV3 cells. Scale bars: 200 μm. **b** The expression of α1,3GT mRNA increased more than 250 times in SKOV3-gal cells compared with parental SKOV3 cells (**P < 0.01). **c** Only about 0.8 % of parental SKOV3 cells showed nonspecific fluorescence. **d** More than 99 % of SKOV3 transfectant expressed α-gal epitopes on the cell surface by flow cytometry

Porcine α1,3GT cDNA was sub-cloned into pCDH-CMV-MCS-EF1-copGFP, α1,3GT recombinant plasmid (Additional file 3: Figure S1). Then using a three plasmid packaging system, the recombinant lentivirus vector expressing porcine α1,3GT gene was constructed and transferred to SKOV3 cells to produce SKOV3-gal cells. Three days after lentivirus infection, GFP signal in SKOV3-gal cells could be observed (Fig. 1a). High expression levels of α1,3GT mRNA were also detected in SKOV3-gal cells by real-time PCR (*p* < 0.01, Fig. 1b), and flow cytometry assay demonstrated that more than 99 % of SKOV3 transfectant expressed α-gal epitopes on the cell surface while only about 0.8 % of parental SKOV3 cells showed nonspecific fluorescence (Fig. 1c and d).

### α-gal epitope expression did not affect the properties of SKOV3-gal cells

**Fig. 2:**
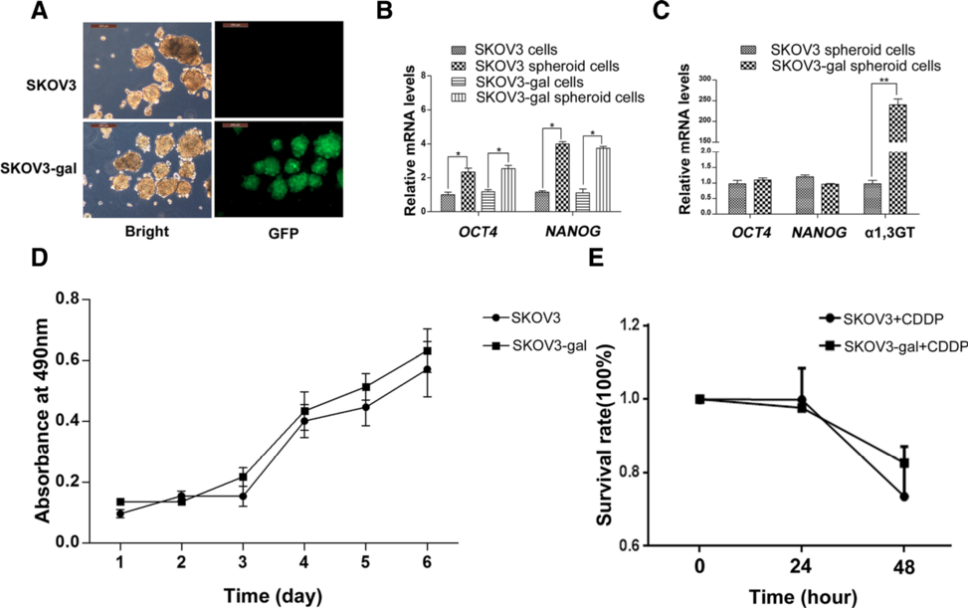
SKOV3-gal spheroid cells could be enriched and the expression of α-gal epitopes did not affect the expression of stem cell markers, cell growth rate, and chemotherapeutic sensitivity. **a** Spheroid cells were formed after 7 days culture with stem cell selection medium. High GFP expression was observed in SKOV3-gal spheroid cells, whereas there was no GFP expression in parental SKOV3 spheroid cells. **b** The expression level of stem cell marker genes *OCT4* and *NANOG* increased significantly in spheroid cells irrespective of α-gal epitope expression (*P < 0.05). **c** SKOV3-gal spheroid cells expressed high levels of a1,3GT (**P < 0.01), but no significant difference was found in transcript levels of *OCT4* and *NANOG* compared with SKOV3 spheroid cells without gal expression. **d** Gal epitope expression did not affect the cell growth rate of SKOV3 cells by MTT assay. **e** SKOV3 cells and SKOV3-gal cells were treated with cisplatin and survival rate of cells was determined by MTT assays. The data were expressed as average absorbance values from five biological replicates and represent means ± standard error (*P* > 0.05)

Previously we reported that ovarian cancer stem-like cells could be enriched by spheroid cell subpopulation under stem cell selection medium [17–19]. Consistent with it, GFP expressing SKOV3-gal cells could be maintained and form non-adherent spheres 7 days after culture with stem cell selection medium (Fig. 2a). The stem cell gene expression level of *NANOG* and *OCT4* increased significantly in both SKOV3 spheroid cells and SKOV3-gal spheroid cells compared with corresponding adherent cells (Fig. 2b). Furthermore, SKOV3-gal spheroid cells expressed significantly higher level of α (1,3) GT (Fig. 2c, **p < 0.01), and no significant difference was found in transcript levels of *OCT4* and *NANOG* compared with SKOV3 spheroid cells.

Both the adherent cells and spheroids of SKOV3-gal cells were the same as the parental SKOV3 cells, by assessment of cell morphology (Figs. 1a and 2a). To investigate whether α1,3GT transfectants could affect the growth of ovarian cancer cells, MTT assays was performed. The results demonstrated that SKOV3-gal cells had the same proliferative capacity as their parental SKOV3 cells (*P* > 0.05, Fig. 2d). In addition, a chemotherapy sensitivity assay was conducted and the results suggested that there was no significant difference of chemotherapy sensitivity to cisplatin between SKOV3 cells and SKOV3-gal cells (Fig. 2e).

### Effectiveness of α-gal epitopes in inducing ovarian cancer cells death by ADCC in vitro

**Fig. 3:**
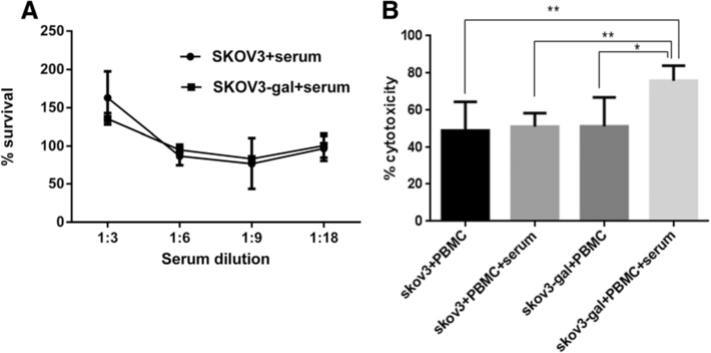
SKOV3-gal cells were killed by human peripheral blood mononuclear lymphocytes (PBMC) from ovarian epithelial cancer patients via antibody dependent cell mediated cytotoxicity (ADCC). **a** The relative cell growth of both SKOV3 cells and SKOV3-gal cells were assayed after culturing with different dilutions of serum supplying anti-gal Abs from ovarian epithelial cancer patients for 24 h. Data represent means ± standard error, *P* > 0.05. **b** The cytotoxicity of PBMC and serum to both SKOV3-gal cells and SKOV3 cells was detected by MTT assays. Data represent means ± standard error, *P < 0.05, **P < 0.01

There is a large amount of anti-Gal which constitutes as much as 1 % of circulating immunoglobulin G in humans [14]. To test the effect of human serum on SKOV3 cells expressing gal epitope, SKOV3-gal cells were incubated in the presence of human serum from EOC patients and cell death was detected by MTT assay. Parental SKOV3 cells were used as negative controls. As shown in Fig. 3a, both SKOV3 cells and SKOV3-gal cells were not killed by serum from EOC patients and there was no significant difference in cell death between SKOV3-gal cells and SKOV3 cells. Intriguingly, however, the addition of both serum and PBMC induced significant cell death of SKOV3-gal cells compared with the parental SKOV3 cells, an effect that could not be reproduced by PBMC alone (Fig. 3b).

### Vaccination with gal expression SKOV3 cells elicited the production of anti-Gal antibodies in vivo

**Fig. 4:**
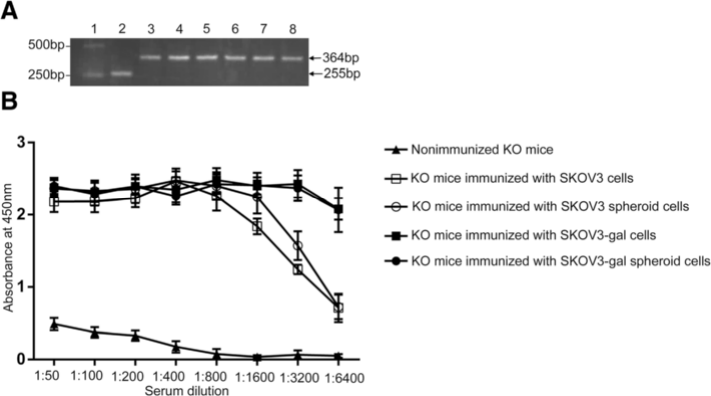
Confirmation of the genotypes of the α1,3GT KO mice and anti-Gal production after immunization. **a** Identification of the genotype of α1,3GT KO mice by PCR. A 255 bp product was detected in wild-type mice, whereas a 364 bp product was produced in α1,3GT KO mice. Lane 1: DL2,000 DNA marker; lane2:genomic DNA from Wild-type mice as template; lanes 3–8:genomic DNA from six donated α1,3GT KO mice as template; **b** Anti-Gal IgG production in α1,3GT KO mice pre-immunized with pig kidney membranes and then immunized with SKOV3 cells, SKOV3 spheroid cells, SKOV3-gal cells, or SKOV3-gal spheroid cells. The anti-Gal IgG production in non-immunized α1,3GT KO mice served as control

To identify the genotype of α1,3GT KO mice, DNA was extracted from the tail of the α1,3GT KO and analyzed by PCR. As shown in Fig. 4a, a 255 bp product was detected in wild-type mice, whereas a 364 bp product was produced in α1,3GT KO mice, which were similar to the results of a previous study [23].

The anti-Gal antibody, as a natural Ab, was negative in non-immunized α1,3GT KO mice. To generate an immune response, the mice were pre-immunized four times with 100 mg of pig kidney homogenates and then immunized subcutaneously three times with SKOV3 cells, SKOV3 spheroid cells, SKOV3-gal cells, or SKOV3-gal spheroid cells. This elicited production of anti-Gal in the immunized mice, quantified by ELISA using synthetic α-gal epitopes coupled to BSA as solid-phase antigen. As shown in Fig. 4b, KO mice immunized with SKOV3-gal cells and SKOV3-gal spheroid cells resulted in extensive production of anti-Gal IgG in the serum, which was higher than that of SKOV3 cell- and SKOV3 spheroid cell-immunized mice serum especially at the serum dilution of 1:3200 and 1:6400 (Fig. 4b, *p < 0.05). In addition, we detected the content of the anti-alpha-gal antibodies in serum of mice immunized with only pig kidney homogenates at the serum dilution of 1:3200 and 1:6400. The results showed that mice that were pre-immunized with pig kidney homogenates, but which were not immunized with SKOV3 nor SKOV3-gal cells, induced the production of anti-alpha-gal antibodies similar to that of SKOV3 cell- or SKOV3 spheroid cell-immunized mice, but lower than that of SKOV3-gal cell- or SKOV3-gal spheroid cell-immunized mice (Additional file 4: Figure S2.).

### The inhibition of tumor growth in mice immunized with α-gal epitope expressing cells

**Fig. 5:**
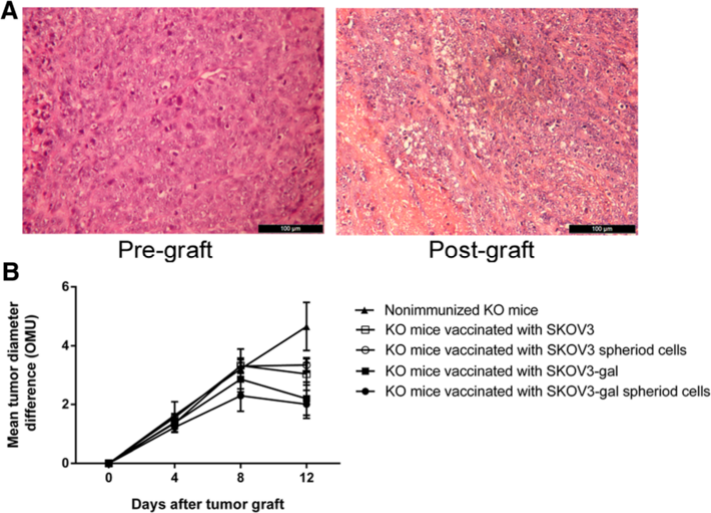
The change of grafted tumor size in α1,3GT KO mice after tumor transplantation. **a** The representative histopathology of pre-graft and post-graft tumor tissues on days 12 by H&E staining. **b** The SKOV3 cells that formed tumors by s.c. transplantation in athymic nude mice were grafted under the kidney capsule of α1,3GT KO mice (*n* = 15 in each group). On days 4, 8, and 12, five mice in each group were sacrificed and the diameters of the tumors were measured *in situ*

To observe the efficiency of the immunization of α-gal epitope expressing cells, the SKOV3 tumors were grafted under renal capsules of KO mice immunized with SKOV3-gal cells. In 150 cases of tumor tissue implantation, 136 cases of grafted tumor continued to grow under renal capsules of KO mice. Representative H&E-stained tumor tissue showed that there was no difference between the histopathology of pre-graft and post-graft tumor tissues, which were categorized as serous adenocarcinoma of Grade 3 and was the original tumor phenotype of the SKOV3 cell line (Fig. 5a). Further, as shown in Fig. 5b, the tumors in non-immunized mice kept growing post graft and increased significantly on days 12 when compared with tumors on days 4 and days 8 (*P* < 0.05). However, although tumors in mice treated with α-gal epitopes expressing SKOV3 cells seemed to keep growing by 8 days post graft, they displayed a slower growth rate than most SKOV3 cell- and SKOV3 spheroid cell-immunized mice. Meanwhile, by 12 days post grafting, the mean tumor diameter decreased in all the immunized mice. Intriguingly, mice immunized with SKOV3-gal cells and SKOV3-gal spheroid cells induced a reduction in tumor size compared with parental SKOV3 cell- or SKOV3 spheroid cell-immunized mice and non-immunized mice (*p* < 0.05).

### α-gal epitope expression induces Abs production against special tumor antigens

**Fig. 6:**
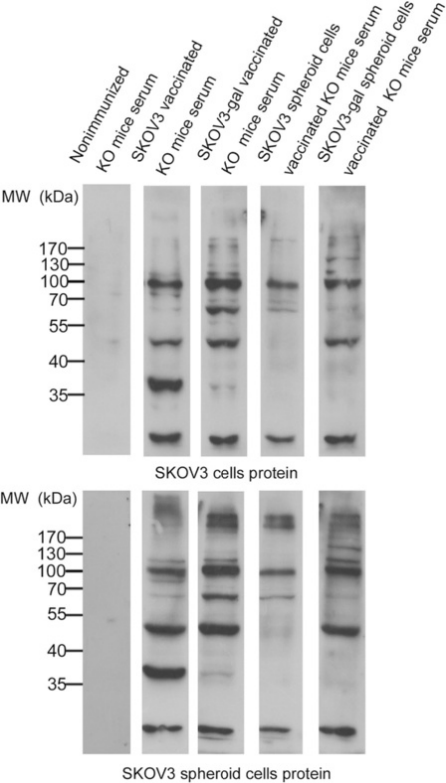
Western blot staining of proteins of SKOV3 cells and SKOV3 spheroid cells with serum from immunized a1,3GT KO mice. MW, molecular weight. Representative data of five experiments with similar results. Lane 1: proteins incubated with serum obtained from non-immunized mice; Lane 2: proteins incubated with serum obtained from SKOV3 cell-immunized mice; Lane 3: proteins incubated with serum obtained from SKOV3-gal cell-immunized mice; Lane 4: proteins incubated with serum obtained from SKOV3 spheroid cell-immunized mice; Lane 5: proteins incubated with serum obtained from SKOV3-gal spheroid cell-immunized mice

To understand the induced Ab response for tumor associated antigens (TAAs), the presence of immunostained proteins of SKOV3 cells and SKOV3 spheroid cells was investigated by western blot with immunized α1,3GT KO mice serum. As shown in Fig. 6, sera from non-immunized KO mice did not contain any Abs that bound to SKOV3 and SKOV3 spheroid cell protein. When reacted with protein from SKOV3 cells, sera obtained from KO mice vaccinated with SKOV3-gal cells displayed several obvious bands (including bands about 170 kDa,150 kDa, 130 kDa,70 kDa, and 55 kDa) which were not reflected in sera obtained from SKOV3 cell-vaccinated mice. Besides, compared with sera obtained from SKOV3 cell-vaccinated mice, the protein bands at 50 kDa,60 kDa, and 100 kDa were thicker when the SKOV3 cell proteins reacted with sera from KO mice vaccinated with SKOV3-gal cells. A similar result was also found comparing sera obtained from KO mice vaccinated with SKOV3-gal spheroid cells and SKOV3 spheroid cells. Sera obtained from KO mice vaccinated with SKOV3-gal spheroid cells also displayed several obvious bands (including bands about 150 kDa and 130 kDa) which were not reflected in sera obtained from SKOV3 cell-vaccinated mice. In addition, bands about 100 kDa and 50 kDa were thicker when SKOV3 proteins were reacted with sera obtained from SKOV3-gal spheroid cell-immunized mice.

However, sera obtained from KO mice vaccinated with SKOV3 cells and SKOV3-gal cells had no significant difference of Ab bound-protein of SKOV3 spheroid cells. Nevertheless, sera obtained from SKOV3-gal spheroid cell-vaccinated KO mice still contained some emerging Abs that bound to proteins (proteins about 150 kDa, 130 kDa, and 50 kDa) which were not reflected in sera obtained from α1,3GT KO mice vaccinated with SKOV3 spheroid cells. The different immune responses induced by sera obtained from KO mice immunized with spheroid cells and parental adherent cells may result from the different gene expression profile of SKOV3 cells and SKOV3 spheroid cells (Additional file 5: Figure S3).

To further elucidate which proteins were contained in the new bands, mass spectrometry was performed. The band at 150 kDa never disappeared no matter whether reacting with protein from SKOV3 cells or SKOV3 spheroid cells, which suggested that immunization with SKOV3-gal spheroid cells could induce Ab production against TAAs from both SKOV3 cells and SKOV3 spheroid cells. So the band about 150 kDa was analyzed. A protein named receptor tyrosine-protein kinase erbB-2 (proto-oncogene c-erbB-2), which was suggested as a prognostic factor in patients with epithelial ovarian carcinoma [27–29], was found abundant in the band.

### siRNAs down-regulated ERBB2 mRNA expression in SKOV3 cells and protein c-erbB-2 expression at the 150 kDa band

**Fig. 7:**
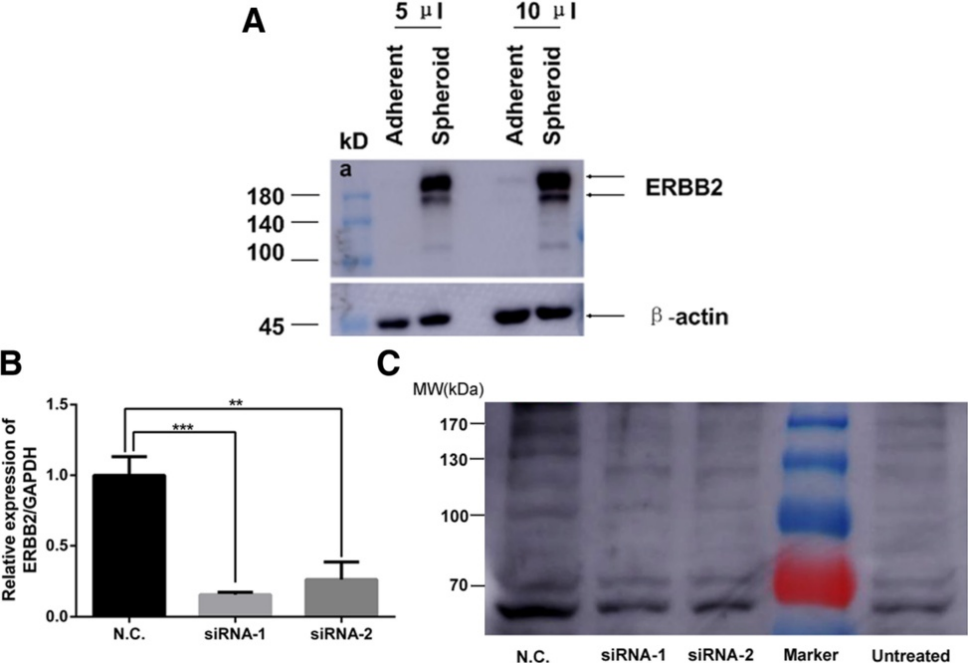
The antibody against protein c-erbB2 could be induced by SKOV3-gal spheroid cell immunization. **a** Western-blot analysis of the lysates of SKOV3 adherent and spheroid cells. 5 μl and 10 μl lysates were loaded and detected with anti-ERBB2 antibody and anti-β-actin antibody. **b** Real-time PCR analysis of ERBB2 mRNA expression 48 h after transfected with siRNAs to knock down ERBB2. **c** Proteins of SKOV3 cells transfected with siRNAs were collected and analyzed for protein expression at 150 kDa after incubation with serum from SKOV3-gal spheroid immunized mice by western blot analysis. A control siRNA that could not target any human mRNA was synthesized as a negative control (N.C. for short). Additionally, SKOV3 cells without transfectants served as untreated control (Untreated for short)

As shown in Fig 7a, anti-ERBB2 antibody could detect two distinct bands in both adherent and spheroid cell lysates of SKOV3 cells. And the abundance of the bands was much higher in spheroid cells than in adherent cells. The molecular weight of one band was about 185 kDa, which should be the full length ERBB2. The molecular weight of the other band was between 180 kDa and 140 kDa, which was speculated by us to be a truncated form of ERBB2 protein of about 150 kDa. RNA interference (RNAi) technology using small interference RNA (siRNA) was used to knockdown ERBB2 mRNA and protein expression. The real-time PCR analysis results showed that the relative level of ERBB2 mRNA in SKOV3 cells transfected with either siRNA-1 or siRNA-2 was significantly lower than negative controls at 48 h after transfection (Fig. 7b). To confirm the interference effect of siRNAs on protein expression of c-erbB-2 and to testify whether c-erbB-2 was the main protein in the 150 kDa band, protein collected from SKOV3 cells transfected with siRNA-1, siRNA-2, or negative control sequence were reacted with serum from SKOV3-gal spheroid cell-immunized mice. As shown in Fig.7b, the 150 kDa band protein expression was decreased after transfection with either siRNA-1 or siRNA-2 (Fig. 7c). This result suggested that the truncated c-erbB-2 (full-length c-erbB-2 was 185 kDa) may exist in the 150 kDa band. And the mice immunized with SKOV3-gal spheroid cells could produce Abs against truncated c-erbB-2.

### Vaccination with SKOV3-gal spheroid cells promoted the production of CD3 + CD4+ T cells in vivo

**Fig. 8:**
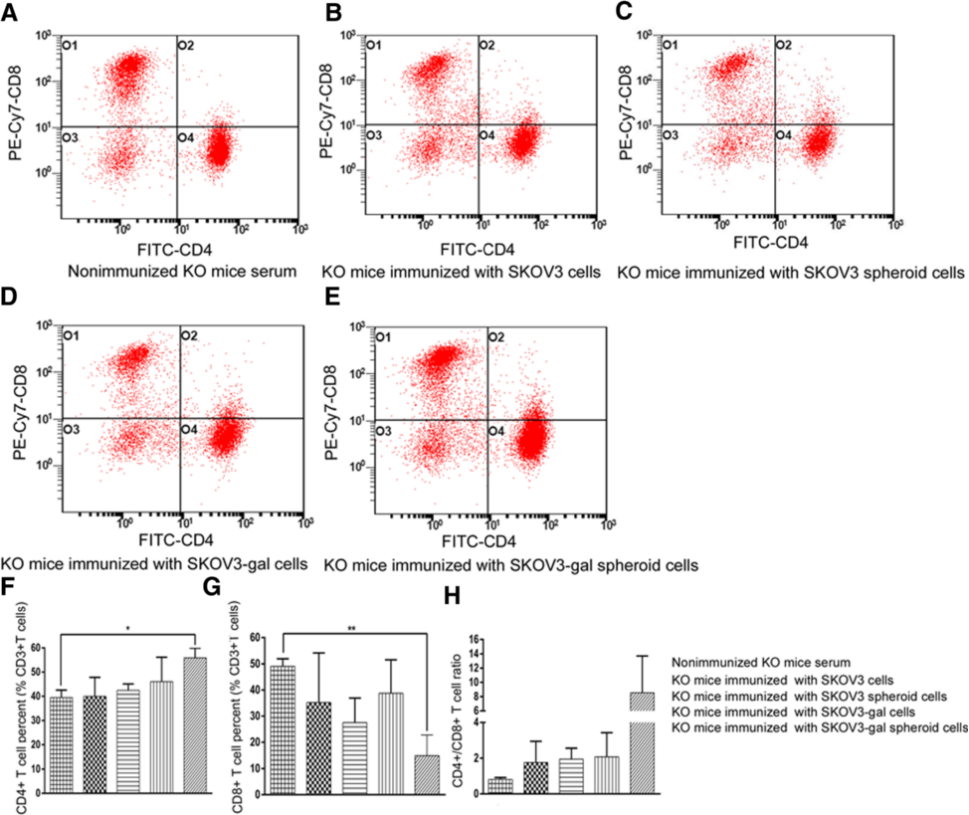
Flow cytometry analysis of levels of CD3 + CD4+ and CD3 + CD8+ T cells in immunized a1,3GT KO mice. Mouse spleen mononuclear cells were isolated by density gradient centrifugation on Ficoll-Paque. And three different colors were used for CD3+, CD4+ and CD8+ membrane markers. Gated CD3 positive events were analyzed for CD4 and CD8 production. The graph of flow cytometry represents data of five experiments with similar results. **a** The distribution of CD3+CD4+ and CD3+CD8+ T cells in spleen from nonimmunized mice. **b** The distribution of CD3+CD4+ and CD3+CD8+ T cells in spleen from mice immunized with SKOV3 cells. **c** The distribution of CD3+CD4+ and CD3+CD8+ T cells in spleen from mice immunized with SKOV3 spheroid cells. **d** The distribution of CD3+CD4+ and CD3+CD8+ T cells in spleen from mice immunized with SKOV3-gal cells. **e** The distribution of CD3+CD4+ and CD3+CD8+ T cells in spleen from mice immunized with SKOV3-gal spheroid cells. **f** The frequency of CD3+CD4+ T-cells. **g** The frequency of CD3+CD8+ T cells. **h** The ratio of CD3+CD4+ CD8- cells to CD3+CD4- CD8+ cells (*P < 0.05, **P < 0.01)

In order to determine whether gal epitope expression on vaccinated cells could activate CD8+ T cells, which become Cytotoxic T cells and destroy tumor cells, or activated CD4+ T cells, which help tumor-specific B cells to produce antitumor antibodies [30], we performed flow cytometry analysis. As shown in Fig. 8, compared with their non-immunized counterparts, mice immunized with SKOV3-gal spheroid cells demonstrated an increased frequency of CD3 + CD4+ T-cells (*P* < 0.05), with a concomitant decreased frequency of CD3 + CD8+ T cells (*P* < 0.01). In addition, the mononuclear cells from the spleen of SKOV3-gal spheroid vaccinated mice displayed a significant increase in the ratio of CD3 + CD4+ CD8- to CD3 + CD4- CD8+ compared with non-immunized mice.

## Discussion

Tumor escape from immune response is considered as the greatest impediment to the application of effective immunotherapy [31]. It was reported that a three-stage process was needed for the generation of effector T cells after vaccination with tumor cell vaccine. Firstly, an effective immune response requires tumor-antigen uptake and processing at the site of injection by APCs. Then APCs migrate into the regional draining lymph nodes, where tumor-specific T cells are activated respectively by TAA peptides presented on MHC class I and class II molecules of APCs. Finally circulation of activated T cells leads to tumor cell destruction [32, 33].

Since the identity of specific TAAs of most types of cancer is not known, the irradiated autologous tumor may be used as a source of vaccinating material. However, tumor cells in cancer patients evolve in a stealthy way lacking identifying markers that label them for uptake by APC, so they can’t be detected by APCs, even in the form of vaccine [34]. It was reported that autologous tumor cell membrane vaccine processed to express α-gal epitopes by incubating ovarian carcinoma membranes prepared from homogenates of freshly obtained tumors with recombinant α1,3GT could successfully induce anti-tumor vaccination *via* the formation of immune complexes by exploiting the natural anti-Gal Ab and its ligand the α-gal epitope [34]. However, it did not seem that all the TAAs were on the membranes of the cancer cells - some were within the cytoplasm of the cancer cells. Therefore, cytoplasmic TAAs may not be targeted to APC when the autologous tumor vaccine consists of only tumor membranes [35]. In addition, CSCs occupy only a small size in the whole tumor population, thus the quantity of ovarian CSCs in the tumor membranes may be too small to induce immune responses against CSCs, which might affect the efficiency of the vaccination [36].

Previous studies showed that ovarian cancer stem-like cells could be enriched by culturing spheroid cells under a serum free stem cell selective system [17–19]. In the present study, we compared SKOV3 spheroid cells and SKOV3 differentiated cells by cDNA microarray and found that SKOV3 spheroid cells have a distinct gene expression profile (Additional file 5: Figure S3). By using this cell model, we obtained intact ovarian cancer stem-like cells expressing α-gal epitopes.

Complement dependent cytolysis (CDC) and ADCC were suggested as the main mechanism that induce xenograft cell destruction and xenograft rejection following the binding of the natural anti-Gal to α-gal epitopes on xenograft cells [37, 38]. More than 90 % of A375 melanoma cell expression of α-gal epitopes was killed when exposed to human serum [39]. Similarly, it was reported that 98 % of α-gal-positive MC38 colon carcinoma cells were killed by media containing human serum [16]. However, Li et al. reported that there is no significant difference in susceptibility to lysis at various concentrations of human serum in three tested human tumor cell lines expressing gal epitope compared with their corresponding parental cells [40]. Consistent with this report, we incubated SKOV3-gal cells with different dilutions of serum obtained from ovarian cancer patients. However, no significant difference was found with regards to sensitivity to human serum-mediated lysis compared with SKOV3 parental cells. It was suggested that the complement regulatory proteins expressed on the tumor cell surface may inhibit CDC [41]. We investigated whether SKOV3-gal cells could be killed by ADCC. To this end, PBMC and serum from ovarian cancer patients were separated and then co-cultured with SKOV3-gal cells. The MTT assay showed that SKOV3-gal cells were killed when culturing with both PBMC and serum, implying that anti-Gal antibody in ovarian cancer patients’ serum can bind with SKOV3-gal cells and induce targeting of tumor cells to APCs.

The efficiency of the SKOV3-gal spheroid cell vaccine was tested in α1,3GT KO mice. Sera obtained from mice vaccinated with SKOV3-gal spheroid cells contained some new Abs that reacted with protein from both SKOV3 cells and SKOV3 spheroid cells. Mass spectrometric analysis demonstrated that the protein reacted with the new Abs including a protein c-erbB-2, which was associated with poor prognosis in cancers such as salivary gland adenocarcinoma, breast cancer, and lung cancer [42–44]. Meanwhile, overexpression of c-erbB-2 proteins corresponded with aggressive characteristics of tumors, such as advanced clinical stage, high grade, presence of vascular invasion, and chemotherapy resistance, suggesting potential as a prognostic marker in patients with epithelial ovarian carcinoma [27–29]. In this study, we found c-erbB-2 proteins at the 150 kDa band by mass spectrometry analysis. Further, the 150 kDa was thinner when the SKOV3 cell protein was collected after SKOV3 cells were transfected with siRNAs to knock down ERBB2 and then incubated with serum from SKOV3-gal spheroid cell-immunized mice. It was reported that the expression of the NH_2_ terminally truncated ErbB2 (95 kDa) in breast cancer correlated with metastatic disease progression compared with the expression of full-length ErbB2 (185 kDa) [45]. So c-erbB2 may also be truncated (about 150 kDa) in ovarian cancer and correlate with metastatic disease progression. Future studies are required to identify and characterize this molecule as novel TAA for immunotherapy of ovarian cancer.

It was determined that vaccination with inactivated influenza virus processed by the use of recombinant 1,3GT to express multiple α-gal epitopes could activate 12 to 13.7 % of CD4+ T-cell, whereas no significant activation was induced by virus lacking α-gal epitopes. Meanwhile, about six fold higher CD8+ T-cell responses were elicited by vaccine expression α-gal epitopes [46]. Herein, we isolated spleen lymphocytes to identify the immune status of the immunized mice, and results showed that spleen lymphocytes from mice immunized with SKOV3-gal spheroid cells had a higher proportion of CD3 + CD4+ T cells, while the proportion of CD3 + CD8+ T cells decreased significantly when compared with non-immunized KO mice. Indeed, MHC class I restricted CD8+ T cells that specifically lyse tumor cells in vitro are frequently measured to document vaccine efficacy and to serve as a surrogate end point in clinical tumor vaccine trials [12, 47, 48]. However, vaccine strategies that directly enhance the priming of tumor-specific CD4+ T-cells have been shown to augment the systemic rejection of MHC class II negative tumors [49, 50]. Recently, a study reported that cancer cells pre-treated with paclitaxel and doxorubicin could enhance cancer-specific CD4+ T cell production and thus induce significant apoptosis and a therapeutic anti-tumor immune response in a mouse model [51]. In this study, CD4+ T cells also mediated significant anti-tumor effector functions in this ovarian CSC vaccination. It seemed that mice immunized with SKOV3-gal cells did not induce a similar immune response as SKOV3-gal spheroid cells did, which may result from the distinct gene expression profile of spheroid cells and adherent cells.

Unfortunately, we failed to establish an ovarian cancer xenograft in the α1,3GT KO mice. 5 × 10^6^ SKOV3 cells, which formed tumors in nude mice, were injected subcutaneously into α1,3GT KO female mice, however, no macroscopic tumor developed. It was reported that human tumor xenografts, including tumors that had been established in serial transplantation in athymic nude mouse hosts or primary surgical tumor tissues, implanted under the renal capsule of normal mice were quantifiable in ocular micrometer units for six days and could retain morphological and functional characteristics of the parent tumors [26, 52]. So we administered injections of SKOV3 cells s.c. into female nude mice to from tumors and then cut them into small pieces (1 mm^3^) to graft under the renal capsules of immunized α1,3GT KO mice. Our results showed that the grafted tumors in non-immunized mice continued to grow, while grafted tumors in mice immunized with all four kinds of vaccine (SKOV3 cells, SKOV3 spheroid cells, SKOV3-gal cells or SKOV3-gal spheroid cells) grew slower than non-immunized mice. Furthermore, xenografts in mice immunized with α-gal expressing cells were smaller at 12 days after grafting when compared with grafts in SKOV3 cell- and SKOV3 spheroid cell-immunized mice.

Whalen and his colleagues [53] had suggested that intra-tumoral injection of α-gal glycolipids is feasible and safe for inducing a protective anti-tumor immune response. It was found to be safe after intra-tumoral injection of 0.1, 1, and 10 mg α-gal glycolipids per tumor. No patients developed clinical signs of toxicity, or any indication of autoimmunity [53]. Accordingly, immunotherapy by synthesis of alpha-gal epitopes on the tumor cells may be safe also, but still needs further study.

## Conclusions

Taken together, buildup of α-gal epitopes on ovarian cancer stem-like cells could activate effective immune responses against TAAs associated with poor prognosis of ovarian cancer and inhibit tumor growth in vivo. Furthermore, approaches using vaccines of α-gal epitope–expressing ovarian cancer stem-like cells are anticipated to induce immune responses against not only differentiated cancer cells but also cancer stem-like cells, and may lead to a cure for ovarian cancer.

## Additional files

Additional file 1: Table S1. PCR primers used to detect gene expression. (DOC 34 kb) Additional file 2: Table S2. siRNA sequences used in this study. (DOC 30 kb) Additional file 3: Figure S1. Construction of recombinant plasmid expressing porcine α1,3GT gene. (A) Restriction enzyme analysis of 1, 3 GT coding sequence. (B) The vector map of pCDH-CMV-MCS-EF1-copGFP. (C) GFP expression in SKOV3 cells after transfection with recombinant plasmid. (D) The expression level of α1,3GT mRNA increased in SKOV3 cells after transfection with recombinant plasmid pCDH-CMV-α1,3GT-EF1-copGFP (***p* < 0.01). (E) α-gal epitopes could be detected in SKOV3 cells by immunofluorescence assay after transfection with recombinant plasmid pCDH-CMV-α1,3GT-EF1-copGFP. Scale bars: C, 200 μm; E, 100 μm. (TIF 733 kb) Additional file 4: Figure S2. Production of anti-Gal antibodies in mice at the serum dilution of 1:3200 and 1:6400. (TIF 396 kb) Additional file 5: Figure S3. Gene expression profile of differentiated cells and spheroid cells. Microarray analysis showed that the gene expression profile was different between SKOV3 cells and SKOV3 spheroid cells. Lane 1: SKOV3 spheroid cells; Lane2: SKOV3 cells. (TIF 153 kb)

## Abbreviations

CSCs: Cancer stem cells
α1,3GT: α(1,3)galactosyltransferase enzyme
SKOV3-gal cells: SKOV3 cells expressing α-gal epitopes
PBMC: Peripheral blood mononuclear lymphocytes
ADCC: Antibody-dependent cell-mediated cytotoxicity
PCR: Polymerase chain reaction
ELISA: Enzyme-linked immunosorbent assay
