# Supplementary material for: Epithelial ovarian cancer stem-like cells expressing α-gal epitopes increase the immunogenicity of tumor associated antigens
Source: BMC Cancer. 2015 Dec 16;15:956. doi: 10.1186/s12885-015-1973-7 (PMC4682262; doi:10.1186/s12885-015-1973-7)
Supplement: Additional file 1: Table S1. — PCR primers used to detect gene expression. (DOC 34 kb) [file 12885_2015_1973_MOESM1_ESM.doc]

**Supplementary Table 1. PCR primers used to detect gene expression**

| Gene symbol |  | Primer sequence |
| --- | --- | --- |
| α-1,3GT-CDS | Forward | GCTCTAGACATGAGGAGAAAATAATGAATG |
|  | Reverse | CGGGATCCTGGAGAAGTAGCCAGAGTAATA |
| α-1,3GT | Forward | GCTGGGTCCTCTGCGTTCCT |
|  | Reverse | TGCGGACTCCTTCCGCCTCT |
| *OCT4* | Forward | GGCCCGAAAGAGAAAGCGAACC |
|  | Reverse | ACCCAGCAGCCTCAAAATCCTCTC |
| *NANOG* | Forward | TTCCTTCCTCCATGGATCTG |
|  | Reverse | TCTGCTGGAGGCTGAGGTAT |
| *18sRNA* | Forward | CGGCGACGACCCATTCGAAC |
|  | Reverse | GAATCGAACCCTGATTCCCCGTC |
| Actin, beta | Forward | ACATCAAGGAGAAGCTCTGCTACG |
|  | Reverse | GAGGGGCGATGATCTTGATCTTCA |
