# Supplementary material for: Epithelial ovarian cancer stem-like cells expressing α-gal epitopes increase the immunogenicity of tumor associated antigens
Source: BMC Cancer. 2015 Dec 16;15:956. doi: 10.1186/s12885-015-1973-7 (PMC4682262; doi:10.1186/s12885-015-1973-7)
Supplement: Additional file 2: Table S2. — siRNA sequences used in this study. (DOC 30 kb) [file 12885_2015_1973_MOESM2_ESM.doc]

S2 Table. siRNA sequences used in this study

| Name | siRNA Sequence 5’→3’ |
| --- | --- |
| siRNA-1 | Sense：GCCUCACAGAGAUCUUGAAUU |
|  | Anti-sense: UUCAAGAUCUCUGUGAGGCUU |
| siRNA-2 | Sense：GGGACGAAUUCUGCACAAUUU |
|  | Anti-sense: AUUGUGCAGAAUUCGUCCCUU |
| N.C. | Sense：UUCUCCGAACGUGUCACGUTT |
|  | Anti-sense: ACGUGACACGUUCGGAGAATT |
