# Supplementary figures and images for: Epithelial ovarian cancer stem-like cells expressing α-gal epitopes increase the immunogenicity of tumor associated antigens
Source: BMC Cancer. 2015 Dec 16;15:956. doi: 10.1186/s12885-015-1973-7 (PMC4682262; doi:10.1186/s12885-015-1973-7)

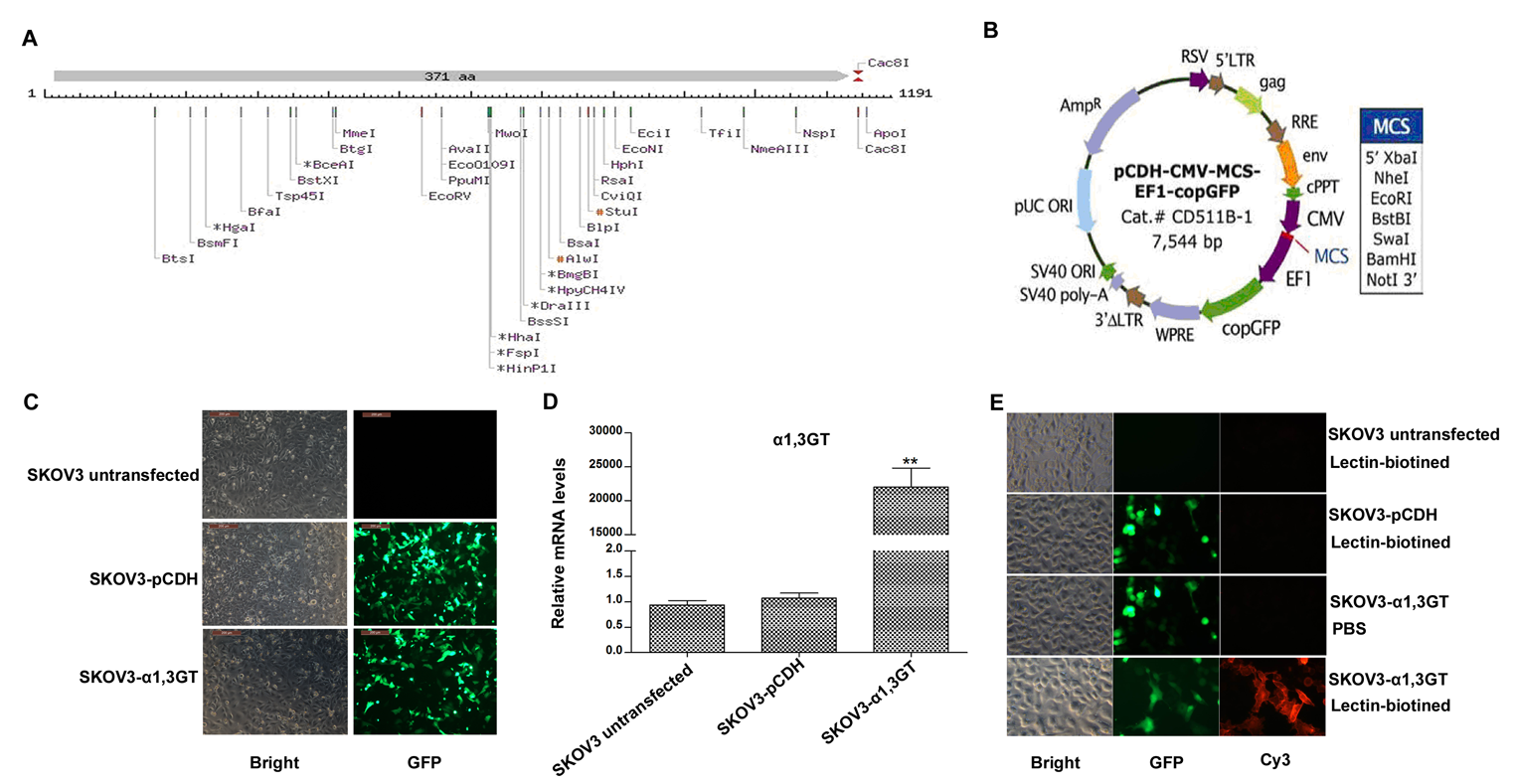

Supplement: Additional file 3: Figure S1. — Construction of recombinant plasmid expressing porcine α1,3GT gene. (A) Restriction enzyme analysis of 1, 3 GT coding sequence. (B) The vector map of pCDH-CMV-MCS-EF1-copGFP. (C) GFP expression in SKOV3 cells after transfection with recombinant plasmid. (D) The expression level of α1,3GT mRNA increased in SKOV3 cells after transfection with recombinant plasmid pCDH-CMV-α1,3GT-EF1-copGFP (**p < 0.01). (E) α-gal epitopes could be detected in SKOV3 cells by immunofluorescence assay after transfection with recombinant plasmid pCDH-CMV-α1,3GT-EF1-copGFP. Scale bars: C, 200 μm; E, 100 μm. (TIF 733 kb) [file 12885_2015_1973_MOESM3_ESM.tif]

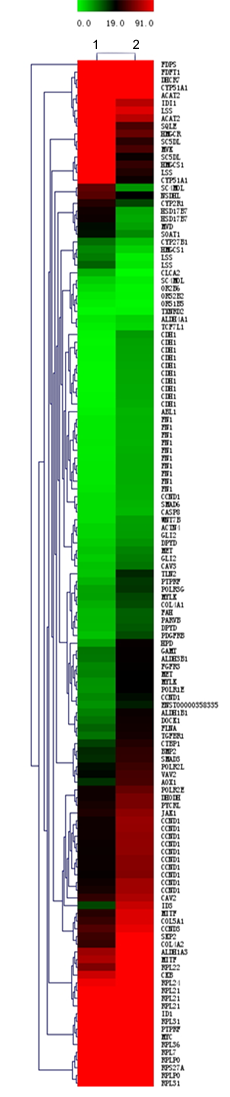

Supplement: Additional file 5: Figure S3. — Gene expression profile of differentiated cells and spheroid cells. Microarray analysis showed that the gene expression profile was different between SKOV3 cells and SKOV3 spheroid cells. Lane 1: SKOV3 spheroid cells; Lane2: SKOV3 cells. (TIF 153 kb) [file 12885_2015_1973_MOESM5_ESM.tif]
